# Interspecies Biofilm Dynamics Among Staphylococci: Inflammatory Contributions to Chronic Rhinosinusitis

**DOI:** 10.1002/alr.70148

**Published:** 2026-03-31

**Authors:** Sintayehu Ambachew, Mahnaz Ramezanpour, Clare M. Cooksley, Sholeh Feizi, Muhammed Awad, Kevin Aaron Fenix, Peter‐John Wormald, Alkis J. Psaltis, Sarah Vreugde

**Affiliations:** ^1^ Adelaide Medical School Faculty of Health and Medical Sciences The University of Adelaide Adelaide Australia; ^2^ The Department of Surgery—Otolaryngology, Head and Neck Surgery University of Adelaide and the Basil Hetzel Institute for Translational Health Research, Central Adelaide Local Health Network South Australia Australia

**Keywords:** biofilm, biomass, chronic rhinosinusitis, *S. aureus*, *S. epidermidis*, *S. lugdunensis*

## Abstract

**Introduction:**

*Staphylococcus* species are frequently isolated from the sinonasal niche of chronic rhinosinusitis (CRS) patients. While *Staphylococcus aureus* is often associated with recalcitrant CRS, *Staphylococcus epidermidis* and *Staphylococcus lugdunensis* are largely deemed commensal. The purpose of this study was to investigate interspecies interactions and how those might influence inflammation and susceptibility to antibiotics.

**Methods:**

Twelve staphylococcal isolates were harvested from six CRS patients, each infected with *S. aureus* and *S. epidermidis*, or with *S. aureus* and *S. lugdunensis*. Bacteria were cultured to allow biofilm formation in direct and indirect interspecies interactions, followed by measuring their biofilm biomass, antibiotic sensitivity, and toxicity and inflammatory potential when applied to human nasal epithelial cells (HNECs).

**Results:**

*S. epidermidis* produced up to 7.4‐fold higher biomass than *S. aureus* in monocultures, with a reduction in *S. epidermidis* biomass under indirect coculture conditions with *S. aureus* biofilm (*p* < 0.05). In contrast, the biofilm biomass values of both *S. lugdunensis* and *S. aureus* were higher under indirect coculture conditions compared to monocultures for 2/3 paired isolates (*p* < 0.05). *S. epidermidis* monocultures and *S. aureus*/*S. epidermidis* cocultures were less toxic to HNECs than *S. aureus* monocultures. *S. aureus* and *S. lugdunensis* monocultures and *S. aureus/S. lugdunensis* cocultures induced interleukin‐6 (IL‐6) and toxicity to a similar extent versus controls. An increased tolerance to amoxicillin was observed for 2/3 *S. epidermidis* biofilm and for 3/3 *S. lugdunensis* biofilm when in indirect contact with *S. aureus* biofilm (*p* < 0.05).

**Conclusion:**

Overall, staphylococcal interactions were highly strain specific, with *S. aureus* influencing the biofilm‐forming capacity and increasing the tolerance to amoxicillin of both *S. epidermidis* and *S. lugdunensis*. *S. epidermidis* but not *S. lugdunensis* could mitigate *S. aureus* induced epithelial cytotoxicity. These findings support the complex nature of interactions among staphylococci with *S. aureus* and potentially *S. lugdunensis* having a pathogenic role and *S. epidermidis* a protective role within polymicrobial biofilms. Our findings have implications for the inflammatory potential and response to therapy of mixed biofilms.

**Abbreviations**
CFU: colony‐forming units
CLSI: Clinical and Laboratory Standards Institute
CoNS: coagulase‐negative staphylococci
CRS: chronic rhinosinusitis
CV: crystal violet
EPOS: European Position Statement
FBS: fetal bovine serum
FnBPs: fibronectin‐binding proteins
GERD: gastro‐esophageal reflux disease
HNECs: human nasal epithelial cells
IL‐6: interleukin‐6
LK: Lund–Kennedy
LM: Lund–Mackay
MALDI‐TOF MS: matrix‐assisted laser desorption ionization‐time of flight mass spectrometry
MBEC: minimum biofilm eradication concentration
MHB: Mueller‐Hinton broth
MIC: minimum inhibitory concentration
MRSA: methicillin‐resistant *S. aureus*
MSA: mannitol salt agar
PBS: phosphate buffer saline
PIA: polysaccharide intercellular adhesin
SNOT‐22: 22‐Item Sino‐Nasal Outcome Test
TSA: tryptone soy agar
TSB: tryptone soy broth

## 1 Introduction

*Staphylococcus aureus* is a Gram‐positive bacterium frequently colonizing our skin or mucous membranes, particularly the nasal mucosal environment [1]. This bacterium is considered an opportunistic pathogen, occasionally found to be resistant to several antimicrobial therapies, notably methicillin and other beta‐lactam antibiotics [2]. *S. aureus* and methicillin‐resistant *S. aureus* (MRSA) are a threat to global public health, causing high mortality and imposing a significant socioeconomic burden [3, 4].

*S. aureus* possesses numerous characteristics that aid in establishing chronic infections in humans. Biofilm formation is a significant feature, in particular in the context of severe recalcitrant chronic rhinosinusitis (CRS) [5]. Previous research revealed strong positive correlations between *S. aureus* biofilm properties such as biofilm biomass, secretion of exoproteins, biofilm metabolic activity, levels of inflammation measured by eosinophil, and total CD4+ T‐cell frequencies. Furthermore, those biofilm properties were positively correlated with subjective and objective disease severity scores in patients with CRS [6]. This bacterium also possesses the ability to express fibronectin‐binding proteins (FnBPs) and clumping factors, which assist in adhesion to surfaces and colonization [7, 8]. Similarly, several *S. aureus* toxins enable them to invade the tissue and cause cytotoxicity, breaching the mucosal barrier [9, 10]. Furthermore, biofilm formation of *Staphylococcus* species promotes the transfer of genetic elements such as plasmids, transposons, and temperate bacteriophages via horizontal gene transfer. Extensive horizontal gene transfer has been revealed during *S. aureus* co‐colonization with coagulase‐negative staphylococci (CoNS) species, and genes associated with virulence, antimicrobial resistance and biofilm formation have been shown to be transferred across such species [11, 12]. These properties contribute to the aggressive nature of *S. aureus* and make it a formidable pathogen.

CoNS including *Staphylococcus epidermidis*, *Staphylococcus lugdunensis*, and *Staphylococcus saprophyticus*, are also frequently found in CRS patients [13, 14]. In recent years, CoNS have been demonstrated to contribute to the pathogenesis of different types of infections, including CRS [15, 16]. Among CoNS, *S. epidermidis* is the most common in CRS patients [16, 17]. This bacterium can produce phenol‐soluble modulins, peptides which are found to aid biofilm formation through polysaccharide intercellular adhesin (PIA) [18, 19]. Similar to *S. aureus*, *S. epidermidis* biofilm is tolerant to the action of antibiotics and host immune responses [20]. However, the formation of biofilm by *S. epidermidis* is also thought to aid its commensal nature in terms of competing with pathogenic bacteria, immune modulation, and maintaining microbial balances [21].

A new member of the CoNS that has been recently identified as an emerging pathogen is *S. lugdunensis*. It has been linked to various infections, including endocarditis, osteomyelitis, urinary tract infections, and skin and soft tissue infections [22]. Certain strains of *S. lugdunensis* have been shown to disrupt the mucosal barrier, cause cytotoxicity, and induce interleukin‐6 (IL‐6) secretion [16]. The pathogenesis of *S. lugdunensis* is reported to be quite similar to that of *S. aureus*, including biofilm formation, adherence to host proteins (fibrinogen, fibronectin), and various toxin secretion [23].

Biofilm is structurally characterized by complex microbial interaction, with bacterial cells embedded in an extracellular matrix composed of polysaccharides, proteins, and nucleic acids [24]. This matrix protects the bacteria from antibiotics and host immune responses and is implicated in chronic infections [24, 25]. Biofilm formation is common in *S. aureus*, *S. epidermidis*, and *S. lugdunensis*, although they differ in their biofilm components. *S. aureus* and *S. epidermidis* predominantly form biofilms through the production of PIA, whereas *S. lugdunensis* is known to produce protein‐rich biofilms that are devoid of PIA. Eradication of biofilm is challenging, and sublethal antibiotic concentrations can exacerbate biofilm formation [26, 27]. The concentration of antibiotics required to eradicate bacterial biofilm is often more than 1000‐fold the concentration that can kill planktonic cells [27, 28].

Antagonistic or synergistic action can occur during microbial interspecies interactions, influencing biofilm formation through the production of extracellular components/factors or quorum‐sensing factors. This might influence disease progression, complications, and treatment outcomes [29]. For instance, in a study by Pammi et al., mixed‐species biofilms involving *S. epidermidis* and *Candida albicans* had increased *S. epidermidis* extracellular DNA  production, in vitro biomass, and in vivo systemic dissemination of *S. epidermidis* [30]. A decreased sensitivity of bacteria to antimicrobials was seen during mixed biofilm growth of *S. aureus* and *Klebsiella pneumoniae* in comparison with mono‐species biofilms [31]. In another study, the susceptibility of *S. aureus* to antimicrobial agents was influenced by the presence of accompanying bacteria [29, 32].

Several studies report on mono‐species bacterial biofilms [33, 34, 35, 36, 37], even though most species grow in polymicrobial biofilm communities in vivo. Thus, a successful therapeutic approach of novel antibiofilm modalities should consider the real‐life situation where biofilms typically consist of mixed‐species communities. Hence, understanding how microorganisms influence each other through direct and indirect interactions and how that impacts their inflammatory properties and response to antibiotics is crucial.

Thus, the aim of this study is to investigate the direct and indirect interaction of *S. aureus* and *S. epidermidis* or *S. lugdunensis* in biofilm form, and the impact of coculture on inducing toxicity and inflammatory cytokine production by the airway mucosa and antibiotic susceptibility.

## 2 Materials and Methods

### 2.1 Ethics Statement and Patients

Ethics approval for the isolation and use of clinical isolates and for the collection of primary human nasal epithelial cells (HNECs) was obtained from Central Adelaide Local Health Network Human Research Ethics Committee (CALHN HREC ref no.: 13604). The diagnostic criteria for CRS were contented by the American Academy of Otolaryngology and Head and Neck Surgery and the European Position Statement (EPOS) on CRS [38, 39]. The severity of CRS was measured based using Lund–Mackay (LM) [40], Lund–Kennedy (LK) [41, 42], and the patient‐reported 22‐Item Sino‐Nasal Outcome Test (SNOT‐22) questionnaire [43]. A self‐reported questionnaire was used to assess the status of asthma, aspirin sensitivity, and gastro‐esophageal reflux disease (GERD).

### 2.2 Growth Conditions of *S. aureus* and *S. epidermidis*

*S. aureus*, *S. epidermidis*, and *S. lugdunensis* were isolated from the same sinonasal niche of six CRS patients. The identity of these isolates was confirmed by matrix‐assisted laser desorption ionization‐time of flight mass spectrometry (MALDI‐TOF MS; Bruker MBT, SA Pathology, Adelaide, Australia). Tryptone soy agar (TSA) and tryptone soy broth (TSB) were utilized to cultivate these strains at 37°C.

### 2.3 Culture of Single‐ and Dual‐Species Biofilm in 96‐Well Plates

Single colonies were selected from overnight cultures and bacterial suspensions (1.0 McFarland standard = 3.0 × 10^8^ CFU/mL, where CFU is colony‐forming units) prepared in 0.9% NaCl, then diluted 1:15 in TSB. For single‐species biofilms, 150 µL/well of diluted culture was dispensed into 96‐well plates (Thermo Fisher Scientific, Nunc A/S, Roskilde, Denmark). For the mixed species, a 1:1 ratio was added (mixing 75 µL of each strain). Plates were incubated at 37°C on a rotating platform (Ratek Instruments Pty Ltd, Boronia, Victoria, Australia) at 70 rpm for 48 h to allow biofilm formation. Negative control wells containing only TSB were included on each plate.

After biofilm formation, the media was removed, plates washed twice using sterile phosphate buffer saline (PBS) to remove nonadherent cells. The biofilm was allowed to air‐dry for 15 min before further assessment of bacterial biomass.

### 2.4 Measurement of Bacterial Biomass

Biofilm biomass was determined by staining with 0.1% crystal violet (CV) solution as described previously [44].

### 2.5 Determination of CFU

The media was removed from 48‐h bacterial biofilms grown in 96‐well plates, washed twice with PBS, and biofilms removed by scraping using sterile pipette tips and resuspended using 180 µL/well of PBS followed by CFU counting as described [44]. To assess CFU/mL in the mixed‐species biofilms, dilutions were plated in parallel onto mannitol salt agar (MSA, Oxoid, Thebarton, South Australia, Australia) and selective CHROMagar Staphylococcus plates (Edwards Group, Narellan, New South Wales, Australia). All plates were incubated overnight at 37°C, and the following day, colonies were counted. A colony count greater than 30 was considered to ensure the accuracy of the CFU/mL calculation. CFU/mL was calculated using the formula: CFU/mL = (number of colonies × dilution factor)/volume plated in mL) and expressed as log CFU/mL. To determine the proportion of *S. aureus* and *S. epidermidis*, or *S. aureus* and *S. lugdunensis* in the mixed species biofilm, the number of pink colonies on the CHROMagar plates (corresponding to *S. aureus*) was subtracted from the total CFUs of both/mixed species (from MSA), which gave the CFUs of *S. epidermidis*, or *S. lugdunensis*. Experiments were repeated three times.

### 2.6 Determination of Minimum Inhibitory Concentration (MIC)

The broth microdilution technique was employed to determine the MIC of amoxicillin and doxycycline against *S. aureus*, *S. epidermidis*, and *S. lugdunensis* following the Clinical and Laboratory Standards Institute (CLSI) guidelines as described previously [45]. A positive control (bacteria without antibiotics) and a negative control (sterile Mueller‐Hinton broth [MHB] only) were also included. The plates were incubated at 37°C for 18–24 h. After incubation, plates were inspected for visible signs of turbidity. A Bio‐Rad microplate reader was used to measure absorbance at 600 nm. The lowest antibiotic concentration showing no visible growth (no turbidity) and at which OD_600_ was ≤ 10% of the growth control and not exceeding the media blank was recorded as the MIC value. The experiment was repeated three times, and the mode of the triplicate assays was used as the final MIC value. MIC breakpoints were interpreted according to the CLSI guidelines (CLSI, 2020).

### 2.7 Indirect Interaction of Dual‐Species Biofilm

Bacterial suspensions in 0.9% NaCl solution (1 McFarland standard = 3.0 × 10^8^ CFU/mL) of each species were prepared as described. *S. aureus*, *S. epidermidis*, or *S. lugdunensis* suspensions were diluted 1:15 in TSB, followed by 500 µL and 100 µL additions to the bottom and top compartments of a Transwell plate (0.4 µm pore size) (Corning Incorporated, Corning, NY, USA), respectively. Plates were incubated at 37°C for 48 h on a rotating platform to allow biofilm formation. After 48 h, the media was carefully removed. Media from the bottom chamber was sampled and plated using sterile cotton swab onto MSA and incubated overnight at 37°C to evaluate the potential of contamination from the upper chamber.

Both compartments were washed twice using PBS and allowed to air dry completely in a sterile environment to allow fixation of the biofilm followed by CV assays as described [44]. The experiment was repeated three times.

### 2.8 Minimum Biofilm Eradication Concentration (MBEC) Assay Using Transwell Plates

Biofilms were prepared in Transwell plates, washed twice with PBS, and treated with 500 µL of amoxicillin or doxycycline added to the bottom chamber and 200 µL to the upper chamber (64 µg/mL in both chambers) or TSB without antibiotics for no‐treatment growth control. Plates were incubated at 37°C for 24 h. Then, biofilm biomass was measured using CV assay as described. Antibiotic effects were evaluated relative to untreated growth controls: monocultures without antibiotics were used as single‐species controls, and cocultures without antibiotics were used as mixed‐species controls. The percentage of reduction in biofilm biomass was calculated based on the growth control biomass value without antibiotics using the following formula: percentage of biomass reduction = [growth control biomass − treated biomass​]/growth control biomass × 100. Experiments were repeated three times.

### 2.9 Growth of HNECS

HNECs were isolated from patients with CRS as described and harvested when they formed 80%–90% confluency [46].

### 2.10 Host–Bacteria Coculture Assay Using Transwell Inserts

HNECs were detached by incubating with 1 mL of 0.05% trypsin for 10 min at 37°C. The trypsin was then neutralized using 5 mL of 10% fetal bovine serum (FBS) in PBS. The cell suspension was centrifuged at 300 rpm for 5 min to pellet the cells. The cells were then counted, resuspended in culture medium, and adjusted to a final concentration of 20,000 cells/mL. Subsequently, HNECs in 500 µL of PneumaCult‐Ex Plus Medium were seeded into the bottom chamber of each well in the collagen‐coated Transwell plates and cultured for 2–3 days to reach 80%–90% confluency. Separately, bacterial strains including *S. aureus* and *S. epidermidis*, or *S. aureus* and *S. lugdunensis* isolated from the same patients, were prepared by adjusting to a 1.0 McFarland standard and diluting 1:15 in TSB. These bacteria were grown for 24 h either as a monoculture or in a 1:1 mixed (coculture) form on the apical chamber of the separate Transwell plates to form biofilm. After confirming epithelial cell confluency, the 24 h‐grown bacterial cultures' biofilms (from separate wells) were transferred to the upper chamber (insert) of the Transwell system containing epithelial cells in the lower chamber. This setup constituted a host–bacteria coculture system. The plates were incubated for 24 h at 37°C in 5% CO_2_, allowing bacterial secreted products to diffuse through the 0.4 µm membrane and interact with the epithelial cells indirectly. After incubation, the supernatant was collected from the lower chamber and used to perform the lactate dehydrogenase (LDH) release assay and IL‐6 enzyme‐linked immunoassay (ELISA) to assess cytotoxicity and the inflammatory response, respectively.

### 2.11 LDH Assay

After 24 h of the host–bacteria coculture, supernatants were collected from the lower chamber and subjected to LDH assay to assess the cytotoxic effects of the bacteria in both monoculture and coculture conditions. The LDH assay was performed using a commercial LDH kit (Promega Corporation, Madison, WI, USA), and the percentage of cell viability was calculated relative to the control group (cells exposed to media only). Relative viability (%) = (absorbance of control group/absorbance of exoprotein treated cells) × 100%. The experiments were repeated three times.

### 2.12 ELISA Assay

IL‐6 levels were determined using ELISA kits (Thermofisher Scientific, Waltham, MA, USA) after collecting the supernatant from the lower chamber of the Transwell plates following the host–bacteria coculture [46]. PneumaCult‐Ex Plus Medium was used as a negative control. The experiments were repeated three times.

### 2.13 Data Analysis

GraphPad Prism version 10.1.0 (316) (GraphPad Software, San Diego, California, USA) was used to analyze the experimental data. The results were described in terms of mean, mean difference ± standard error of mean (MD ± SEM), and percentages. One‐way ANOVA was employed to compare among the groups, followed by Tukey's post hoc test. A *T*‐test was also used for comparisons between two groups. A *p*‐value < 0.05 was regarded as statistically significant.

## 3 Results

### 3.1 Sociodemographic Characteristics of the Patients

A total of 12 staphylococci were collected from six CRS patients. These included three paired *S. aureus* and *S. epidermidis* and three paired *S. aureus* and *S. lugdunensis*, with each pair isolated from the same sinonasal niche of individual patients. These six patients were selected because of the coinfection status they displayed. According to Toma & Hopkins SNOT‐22 criteria of symptom stratification strategy, two patients had severe CRS status, whereas the remaining patients had mild/moderate CRS [47] (Table 1).

**TABLE 1:** Sociodemographic characteristics of the patients.

Patients	Clinical isolates	Bacterium	CRS status	Age	Gender	GERD	Aspirin sensitivity	SNOT‐22	LK	LM
Patient 1*	C1102 (SA1)	*Staphylococcus aureus*	CRSsNP	32	Male	Yes	No	60	4	6
	C1103 (SE1)	*Staphylococcus epidermidis*								
Patient 2	C1109 (SA2)	*S. aureus*	CRSsNP	79	Male	No	No	41	2	8
	C1110 (SE2)	*S. epidermidis*								
Patient 3	C1112) (SA3)	*S. aureus*	CRSsNP	86	Female	No	Yes	31	4	8
	C1113 (SE3)	*S. epidermidis*								
Patient 4*	C1072 (SA4)	*S. aureus*	CRSwNP	68	Male	No	No	76	3	8
	C1073 (SL4)	*Staphylococcus lugdunensis*								
Patient 5	C880 (SA5)	*S. aureus*	CRSsNP	24	Male	No	No	40	1	3
	C1075 (SL5)	*S. lugdunensis*								
Patient 6	C877 (SA6)	*S. aureus*	CRSsNP	27	Female	No	No	50	3	3
	C1080 (SL6)	*S. lugdunensis*								

Abbreviations: CRSsNP, chronic rhinosinusitis without nasal polyps; CRSwNP, chronic rhinosinusitis with nasal polyps; GERD, gastro‐esophageal reflux disease; LK, Lund–Kennedy; LM, Lund–Mackay score; SA1–SA6, *S. aureus* isolated from Patients 1–6; SE1–SE3, *S. epidermidis* isolated from Patients 1–3; SL4–SL6, *S. lugdunensis* isolated from Patients 4–6; SNOT‐22: 22‐Item Sinonasal Outcome Test.

* Severe patients (SNOT‐22 scores > 50).

### 3.2 Direct Interactions Between *S. aureus* and *S. epidermidis* or *S. lugdunensis* Vary Among Strains

**FIGURE 1:**
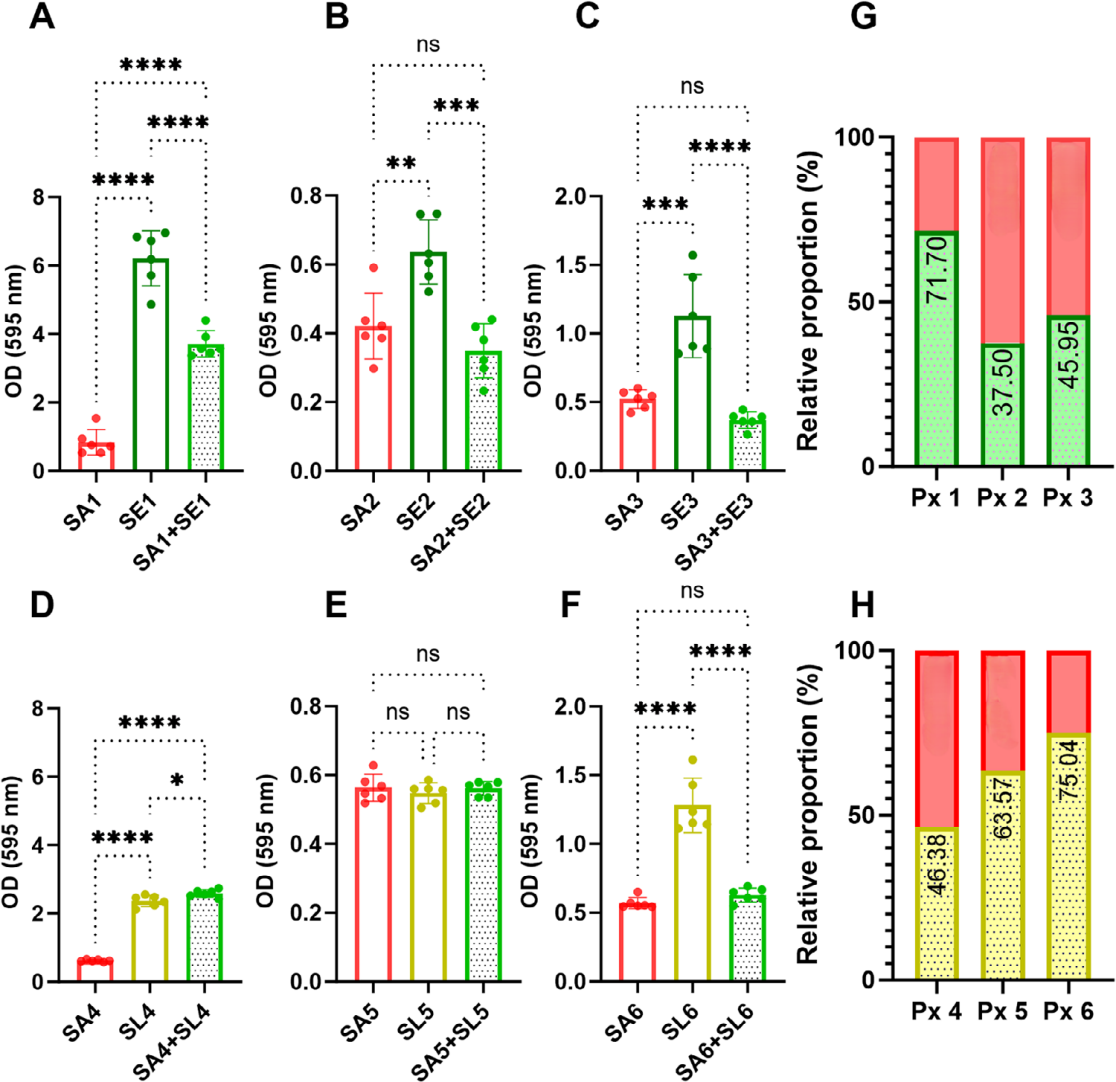
Biofilm biomass value of Staphylococcus aureus (SA), Staphylococcus epidermidis (SE), and *Staphylococcus lugdunensis* (SL) in monoculture and coculture. Biofilm biomass measured in OD (595 nm) for SA, SE, and SL grown in monocultures and cocultures (SA + SE; SA + SL) for Patients 1–3 (panels A–C) and Patients 4–6 (panels D–F). (G and H) Relative proportion of CFU count in a mixed biofilm culture of (G) SA (red) and SE (green, in percentage of total CFU) among isolates from three patients (Px1–Px3), (H) SA (red) and SL (yellow, in percentage of total CFU) among isolates from three patients (Px4–Px6). Results represented as mean ± SEM. *p ≤ 0.05, **p ≤ 0.01, ***p ≤ 0.001, ****p ≤ 0.0001; ns: not statistically significant, one‐way ANOVA followed by Tukey's multiple comparisons test. *N*= 6 replicates.

To investigate whether direct interactions among paired isolates influence their biofilm forming capacity, we compared the biofilm biomass and CFU of strains when grown individually or in coculture. Among the isolates from the different patients, *S. epidermidis* from patient 1 formed the highest biomass biofilms, which was 7.43‐fold higher than the corresponding *S. aureus* biofilm and significantly higher than the mixed *S. epidermidis/S. aureus* biofilms (*p* < 0.05). The biofilm biomass of *S. epidermidis* from Patients 2 and 3 was up to 2.16‐fold higher than the corresponding *S. aureus* biofilms and significantly higher than the mixed *S. epidermidis*/*S. aureus* biofilms (*p* < 0.05). *S. aureus* biofilm biomass was lower than the mixed *S. epidermidis*/*S. aureus* biofilm biomass for Patient 1 only (*p* < 0.001) (Figure 1A–C). These findings were in line with CFU counts for Patient 1, where *S. epidermidis* exhibited high mean CFU counts compared to *S. aureus* monocultures and coculture conditions (*p* < 0.05). In coculture, an average of 71.7% of CFUs were identified as *S. epidermidis* with the remaining 28.3% identified as *S. aureus*. However, for Patients 2 and 3, there was no significant difference in CFU counts of *S. aureus* and *S. epidermidis* biofilm grown in monoculture and coculture conditions (*p* > 0.05) and the proportion of *S. aureus*/*S. epidermidis* in mixed cultures were 62.5% and 54.05% for *S. aureus* for Patients 2 and 3, respectively (Figure 1; Figure S1).

*S. lugdunensis* exhibited up to 3.8‐fold more biofilm biomass than the corresponding paired *S. aureus* in monoculture for Patients 4 and 6 (*p* < 0.05), respectively, and a 2.03‐fold increase to that of the mixed *S. aureus*/*S. lugdunensis* biofilm for Patient 6 (*p* < 0.05). Further, the mixed *S. aureus*/*S. lugdunensis* formed 4.16‐fold more biofilm biomass than *S. aureus* monocultures for Patient 4 (*p* < 0.05), while no significant differences were observed among monocultures and cocultures for Patient 5 (*p* > 0.05) (Figure 1D–F). CFU counts were consistently higher in *S. lugdunensis* biofilms compared to *S. aureus* biofilms for all three pairs and to *S. aureus*/*S. lugdunensis* mixed biofilms for Patient 4 only (*p* < 0.05). In cocultures, between 46.3% and 75.04 % were identified as *S. lugdunensis* (Figure 1; Figure S1).

### 3.3 *S. aureus* Biofilm Secreted Products Can Reduce *S. epidermidis* Biofilm Biomass and Increase *S. lugdunensis* Biofilm Biomass in Some Cases

**FIGURE 2:**
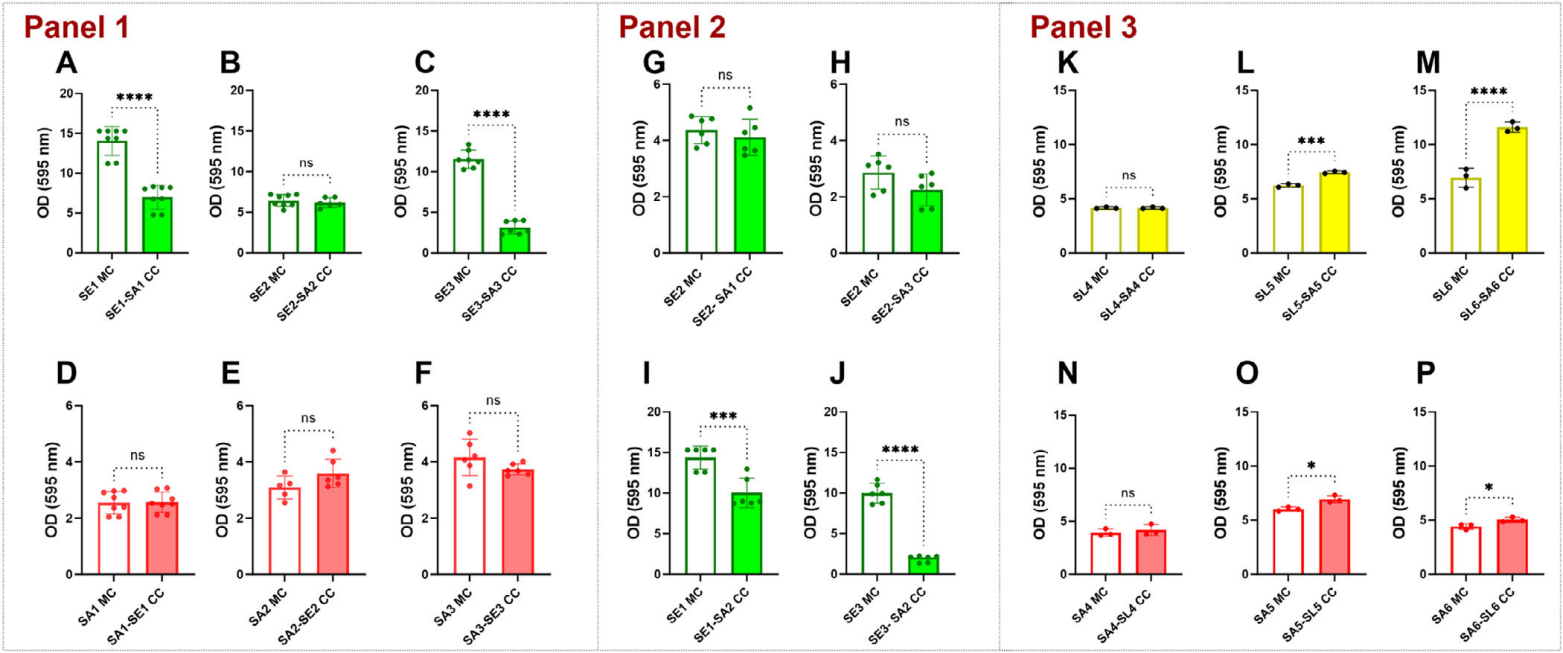
(Panel 1) *Staphylococcus aureus* (SA) secreted factors suppress *Staphylococcus epidermidis* (SE) biofilm formation. Biomass measured by crystal violet with results shown in OD (595 nm) for SE (A–C) and SA (D–F) isolates from Patients 1–3 grown in monoculture (MC) or coculture (CC) using Transwell assays. SE was cultured in the bottom (A–C) or top (D–F) chamber, with SA in the reciprocal position. (Panel 2) Cross‐patient cocultures of SA and SE (G–J) showing SE biomass in the bottom chamber: (G) SE2‐SA1, (H) SE2‐SA3, (I) SE1‐SA2, (J) SE3‐SA2. SE was grown alone (MC) or with SA in the top chamber (CC). (Panel 3) SA secreted products enhance *Staphylococcus lugdunensis* (SL) biofilm formation. Biomass for SL (K–M) and SA (N–P) isolates from Patients 4–6 in MC or CC conditions. SL was cultured in the bottom (K–M) or top (N–P) chamber with SA in the reciprocal position. Data were analyzed using the *T*‐test, results represented as mean ± SEM. *p ≤ 0.05, ***p ≤ 0.001, ****p ≤ 0.0001; ns: not statistically significant.

We then wanted to investigate whether secreted products of one species play a role in biofilm formation of the other. To do this, we established *S. aureus* and *S. epidermidis* or *S. aureus* and *S. lugdunensis* into biofilms separated by a Transwell membrane with isolates each time isolated from the same or from different patients. No contamination of any of the species grown in the upper chamber was identified in the media of the lower chamber, indicating any effects on the biofilm biomass of the presence of the alternate species would be due to indirect effects. The results indicated that *S. epidermidis* biofilms from Patients 1 and 3 were reduced in the presence of *S. aureus* biofilms compared to *S. epidermidis* biofilm grown in monoculture (*p* < 0.0001) while *S. aureus* biofilms from all three patients were similar in the presence or absence of *S. epidermidis* biofilms from those same patients (*p* > 0.05) (Figure 2, Panel 1).

To evaluate whether host‐adaptation of the isolated bacteria might play a role in this effect, *S. aureus* biofilm from Patient 2 was indirectly cocultured with *S. epidermidis* biofilms from Patients 1 and 3, and *S. epidermidis* biofilm from Patient 2 was cocultured with *S. aureus* from Patients 1 and 3. Results showed that *S. epidermidis* biofilm biomass values from Patients 1 and 3 were reduced in the presence of *S. aureus* biofilm from Patient 2 in comparison to *S. epidermidis* biofilms grown in monoculture (*p* < 0.001). Further, *S. epidermidis* biofilm biomass from Patient 2 showed no change in biofilm biomass value, whether *S. aureus* biofilms from Patients 1 and 3 were present or not (*p* > 0.05) (Figure 2, Panel 2). These findings indicate that *S. epidermidis* from Patient 2 is not affected by *S. aureus* while *S. epidermidis* from Patients 1 and 3 have a reduced biofilm forming capacity when grown in indirect contact with *S. aureus* biofilm, regardless on the origin of *S. aureus* (Figure 2, Panel 2).

For indirect interactions between *S. aureus* and *S. lugdunensis*, the biofilm biomass values of single species isolates for Patient 4 showed no significant difference with the coculture condition for both species (*p* > 0.05). In contrast, for Patients 5 and 6, the biofilm biomass values of *S. lugdunensis* and *S. aureus* were higher under coculture conditions compared to monoculture (*p* < 0.05) (Figure 2, Panel 3). However, there was no significant difference in biofilm biomass between monoculture and coculture conditions for any of the isolates when those were isolated from different patients (*p* > 0.05) (Figure S2). These results indicate that at least for some *S. lugdunensis* and *S. aureus* strains, host adaptation might play a role in whether they influence each other's biofilm forming capacity.

### 3.4 *S. epidermidis* but Not *S. lugdunensis* Partially Mitigates the Cytotoxic Effects of *S. aureus*

**FIGURE 3:**
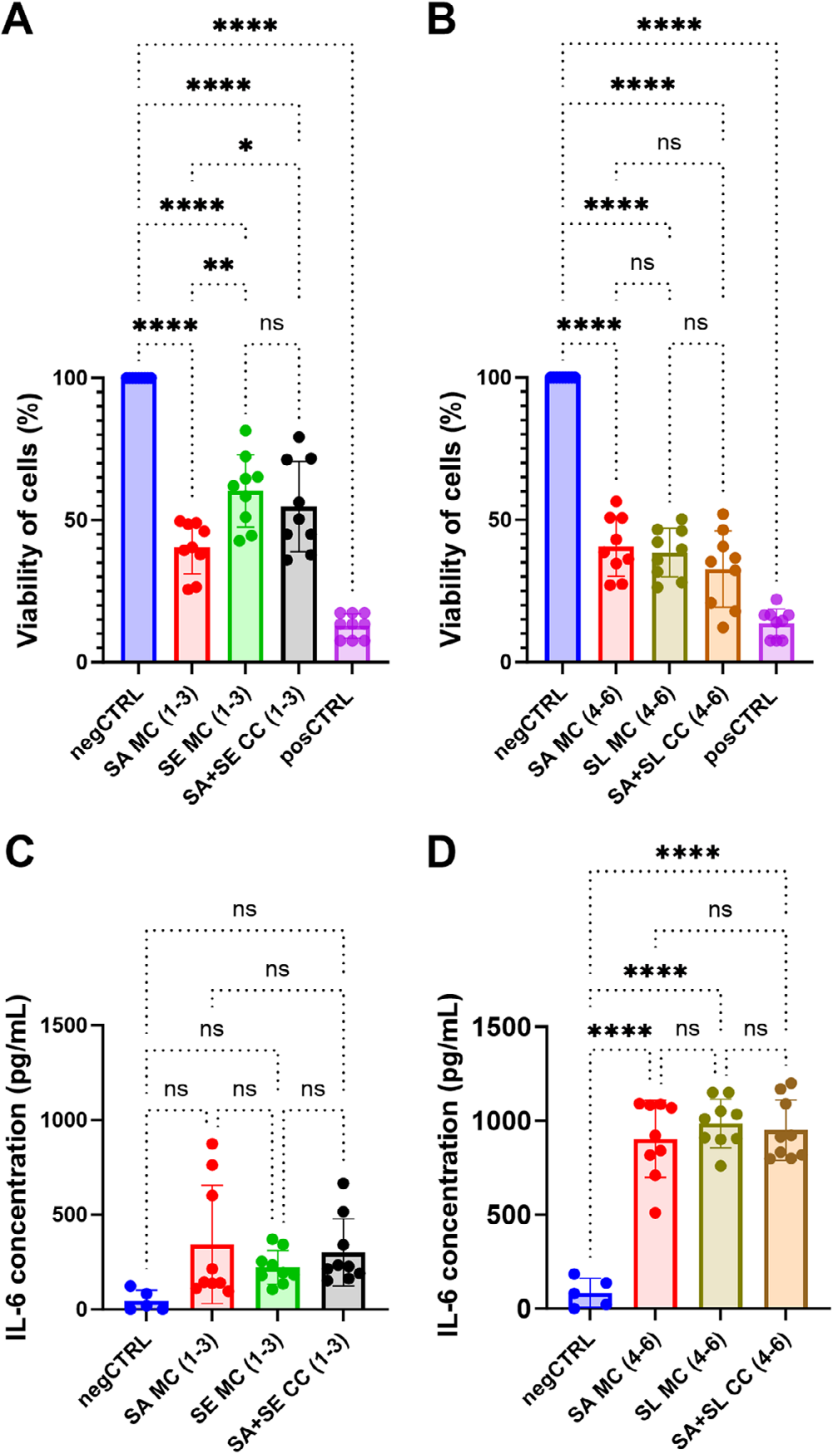
Toxicity and induction of IL‐6 in HNECs following exposure to *Staphylococcus aureus* (SA), *Staphylococcus epidermidis* (SE), and *Staphylococcus lugdunensis* (SL) monocultures (MCs) and direct cocultures (CCs). (A, B) Viability of cells (% to normalized control), (C, D) IL‐ 6 levels (pg/mL) after exposure to biofilm forms of SA, SE, and SL in MC and CC: (A, C) shows isolates from Patients 1–3, while (B, D) shows isolates from Patients 4–6. Data were analyzed using one‐way ANOVA followed by Tukey's multiple comparisons test and are presented as mean ± SEM. *p ≤ 0.05, **p ≤ 0.01, ****p ≤ 0.0001; ns: not statistically significant; *N* = 3 replicates.

We tested the cytotoxic effects and induction of IL‐6 of *S. aureus*, *S. epidermidis*, and *S. lugdunensis* biofilms in monocultures and direct cocultures. *S. aureus* monocultures were more toxic to HNECs than *S. epidermidis* monocultures and *S. aureus*/*S. epidermidis* cocultures (*p* < 0.05). No significant difference was observed in the mean IL‐6 levels for HNECs exposed to *S. aureus* monoculture, *S. epidermidis* monoculture, and *S. aureus/S. epidermidis* mixed biofilms compared to control (*p* > 0.05). Conversely, *S. aureus* and *S. lugdunensis* monocultures as well as *S. aureus*/*S. lugdunensis* cocultures showed similar increased toxicity and IL‐6 induction of HNECs compared to control (*p* < 0.05) (Figure 3).

### 3.5 Evaluation of *S. aureus*, *S. epidermidis*, and *S. lugdunensis* Susceptibility to Amoxicillin and Doxycycline in Planktonic Forms

The susceptibility of *S. aureus*, *S. epidermidis*, and *S. lugdunensis* strains to amoxicillin and doxycycline, commonly used antibiotics to treat CRS [48], was tested employing the broth microdilution method. Based on their MIC values, all isolates were found to be susceptible to both antibiotics according to EUCAST breakpoints and CLSI guidelines (Table S1).

### 3.6 The Presence of *S. aureus* Enhances *S. epidermidis* and S. lugdunensis Biofilm Tolerance to Amoxicillin

**FIGURE 4:**
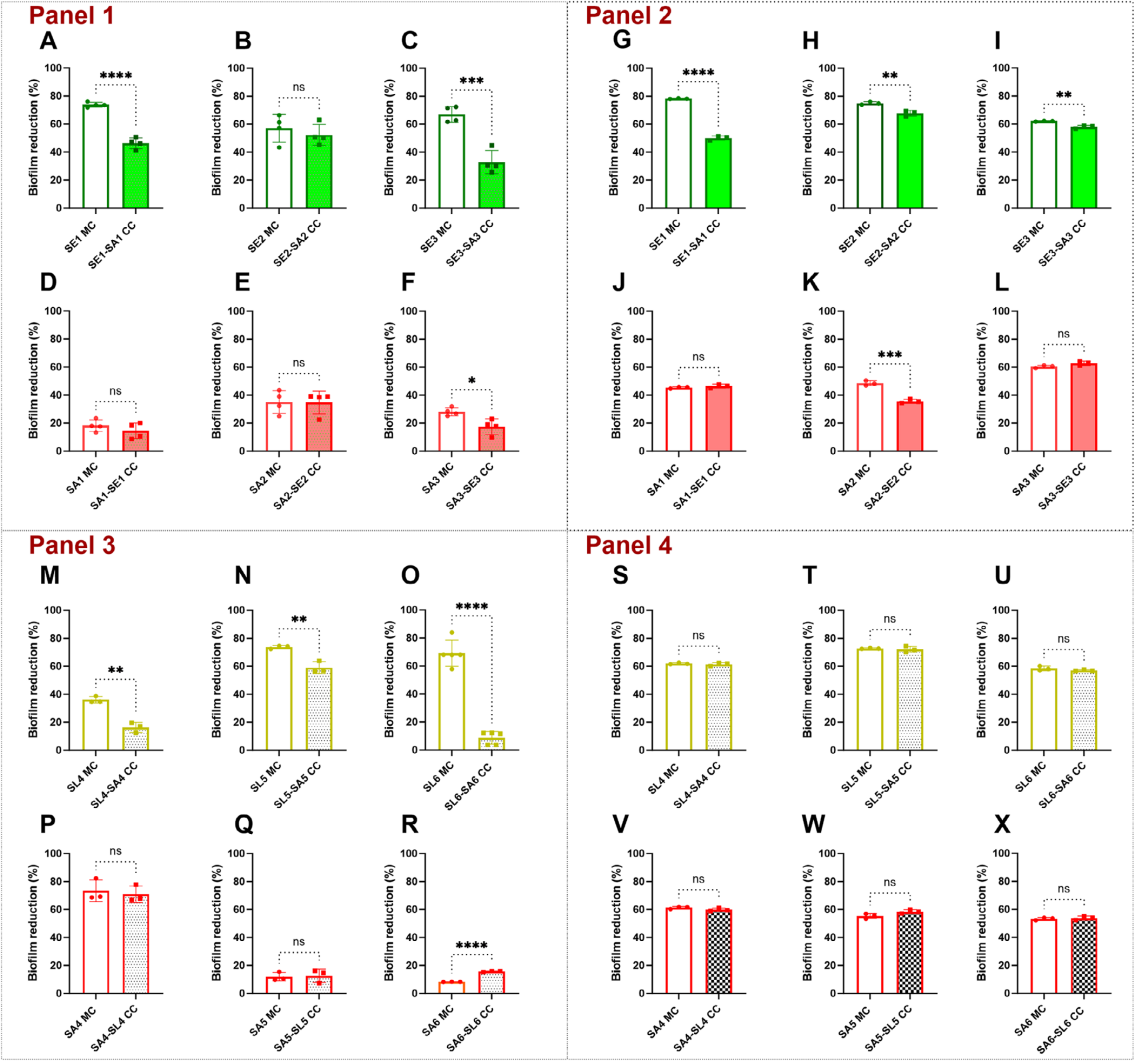
Sensitivity to amoxycillin and doxycycline of *Staphylococcus aureus* (SA), *Staphylococcus epidermidis* (SE), and *Staphylococcus lugdunensis* (SL) biofilms in monocultures and indirect cocultures. Biofilm reduction (% compared to untreated control) of SA, SE, and SL in monoculture (MC) and coculture (CC) following antibiotic treatment in Transwell assays. (Panels 1 and 3) Reduction in biofilm biomass (%) after 64 µg/mL amoxicillin treatment for SE (A–C) and SA (D–F) isolates from Patients 1–3, SL (M–O) and SA (P–R) isolates from Patients 4–6. (Panels 2 and 4) Reduction in biofilm biomass (%) after 64 µg/mL doxycycline treatment for SE (G–I) and SA (J–L) isolates from Patients 1–3, SL (S–U) and SA (V–X) isolates from Patients 4‐6. SE was cultured in the bottom (A–C, G–I) or top (D–F, J–L) chamber, with the corresponding SA occupying the reciprocal position. SL was cultured in the bottom (M–O, S–U) or top (P–R, V–X) chamber, with the corresponding SA occupying the reciprocal position. Biomass values represent species grown in the bottom chamber. The percentage of biofilm reduction was calculated as (Control OD − Treated OD)/Control OD × 100, where untreated biofilms served as controls. Data are presented as mean ± SEM (unpaired *t*‐test; *p* ≤ 0.05, *p ≤ 0.01, **p ≤ 0.001, ***p ≤ 0.0001, ****p ≤ 0.0001; ns = not statistically significant).

We then aimed to determine if the indirect interactions between *S. aureus* and *S. epidermidis* or *S. aureus* and *S. lugdunensis* biofilm affected their response to 64 µg/mL amoxicillin. The results indicate that *S. aureus* biofilm was less sensitive to amoxicillin when *S. epidermidis* was present compared to its monoculture for Patient 3 only (*p* < 0.05). Furthermore, *S. epidermidis* biofilm was less sensitive to amoxicillin when grown in the presence of *S. aureus* biofilm for Patients 1 and 3 (*p* < 0.05) (Figure 4, Panel 1).

Treatment of biofilms with 64 µg/mL doxycycline indicated *S. aureus* biofilm was less sensitive when *S. epidermidis* was present, compared to its monoculture for Patient 2 only. In contrast, *S. epidermidis* biofilm was less sensitive to doxycycline when *S. aureus* was present compared to its monoculture across the three patients (*p* < 0.05) (Figure 4, Panel 2).

*S. lugdunensis* biofilms were less sensitive to amoxicillin when grown in the presence of *S. aureus* than in monoculture for all three patients (*p* <  0.05). Conversely, *S. aureus* biofilms were more sensitive to amoxicillin when grown in the presence of *S. lugdunensis* compared to *S. aureus* monocultures for Patient 6 only, *p* < 0.0001 (Figure 4, panel 3).

Following doxycycline treatment, no significant difference in average biofilm biomass reduction was observed for either *S. aureus* or *S. lugdunensis* between monoculture and coculture conditions in isolates from all three patients (*p* > 0.05) (Figure 4, Panel 4).

## 4 Discussion

In this study, the direct and indirect interaction of *S. aureus* and *S. epidermidis* or *S. lugdunensis* were investigated with respect to their influence on biofilm forming capacity, susceptibility to antibiotics, toxicity, and induction of IL‐6 in HNECs. Our findings indicate that both *S. epidermidis* and *S. lugdunensis* have higher biofilm forming capacity than *S. aureus* in the majority of paired isolates. *S. epidermidis* biofilm biomass values were reduced in indirect cocultures with *S. aureus* biofilms, while both *S. lugdunensis* and *S. aureus* biofilm biomass values were increased in cocultures. Further, enhanced amoxicillin tolerance in both *S. epidermidis* and *S. lugdunensis* biofilms was observed during coculturing these bacteria with *S. aureus*, suggesting cooperative effects in polymicrobial biofilms to help resist antibiotics. Lastly, direct interactions of *S. aureus* and *S. epidermidis* but not *S. aureus* and *S. lugdunensis* biofilm reduced *S. aureus* biofilm mediated cytotoxic effects.

The present study demonstrated that *S. epidermidis* produced more biomass than the paired isolates of *S. aureus*. Similar observations have been reported by Águila‐Arcos et al. and Mirzaei et al., who found that *S. epidermidis* tends to be a stronger biofilm former than *S. aureus* [49, 50]. The high production of important extracellular matrix components, such as PIA, and proteins like accumulation‐associated protein, extracellular matrix‐binding protein, and FnBPs has been linked to *S. epidermidis’* exceptional capacity to produce biofilms [51]. Furthermore, phenol‐soluble modulins, extracellular DNA release, and the participation of several regulatory systems all support *S. epidermidis* robust biofilm phenotype [51, 52]. However, the biofilm biomass values of *S. epidermidis* monocultures exhibited greater variability among strains, probably due to variation in biofilm associated genes among strains compared to *S. aureus* monocultures. In the mixed biofilms, the total biomass of *S. aureus* and *S. epidermidis* was lower than that of *S. epidermidis* monocultures. The CFU count revealed that, in mixed biofilms, *S. aureus* tended to dominate the population, except in patient 1, where *S. epidermidis* was the predominant biofilm‐forming species. This might be related to changes in the cell number, metabolic activities, and biofilm‐related genes [53, 54, 55]. Notably, this patient also had high SNOT‐22 scores, suggesting the potential role of *S. epidermidis* biofilm contributing to the inflammation in this patient. While further analysis in more isolates is required, these findings indicate that *S. epidermidis* might at least in some patients not act as an innocent bystander but might contribute to inflammation.

During the direct interaction between *S. lugdunensis* and *S. aureus*, the biofilm biomass exhibited a dynamic pattern. Across isolates, some mixed biofilms showed increased biomass compared to their respective monocultures, others showed a reduction, or no significant difference was observed. Like *S. epidermidis*, the biomass of *S. lugdunensis* varied considerably among different strains. However, the paired *S. aureus* isolates from the same patients displayed relatively consistent or lower variability in their biofilm biomass values. The variable biofilm biomass among different strains of *S. epidermidis* and *S. lugdunensis* suggests these species biofilm formation is strain‐dependent, which indicates individual isolates might have different genetic and phenotypic capacities for adhesion, matrix production, and quorum‐sensing regulation [56]. *S. aureus* shows less strain‐dependent variability, which might be due to a stable biofilm‐forming phenotype driven by conserved genetic determinants (e.g., *icaADBC*, *sarA*, *agr*) and suggestive of less influence by interspecies or environmental factors, thus having a more predictable virulence capacity [57, 58].

In the indirect interaction assays, *S. aureus* was found to reduce the biofilm formation of *S. epidermidis* in 2/3 isolates. Interestingly, this appeared to be due to the inherent properties of those specific *S. epidermidis* strains, as indirect interactions with non‐matched *S. aureus* resulted in similar reductions in the biomass values of those strains. This might be due to the release of *S. aureus* extracellular molecules (secreted enzymes and metabolites), quorum sensing molecules, eDNA, and the secretion of toxins and exoenzymes that could enhance the dispersal of *S. epidermidis* [59]. Of note, despite the reduction in *S. epidermidis* biomass observed, these same *S. epidermidis* strains were found to be more tolerant to antibiotics when in indirect contact with *S. aureus*. This apparent discrepancy might be because of the notion that antibiotic susceptibility is not determined solely by total biomass but also by cellular physiology [60]. For example, soluble factors secreted by *S. aureus* might alter *S. epidermidis* into a metabolically slow‐growing, stress‐adapted state, which is associated with antibiotic tolerance as suggested in a study by Vandecandelaere et al. [61]. Nonetheless, in our study, not all *S. epidermidis* isolates were affected; notably, the *S. epidermidis* isolate from Patient 2 exhibited resistance to the inhibitory effect of all *S. aureus* strains tested, and its tolerance to amoxicillin was similar to that of its monocultures. Similarly, the biofilm biomass value of *S. aureus* was not influenced by *S. epidermidis* during indirect interaction. This might be due to the presence of conserved genetic determinants in these strains that make them nonresponsive to interactions from the alternate species. More in‐depth genomic analysis is required to investigate this further.

In contrast, both *S. aureus* and *S. lugdunensis* pairs from the same patient but not from different patients showed an increase in biofilm biomass during indirect cocultures for 2/3 patients relative to their respective monocultures. These findings indicate that niche‐adaptation found to be important in chronic *S. aureus* colonization, might play a role in the coordinated reciprocal action on enhancing both species’ biomass [62]. One potential mechanism that could explain such coordinated behavior is quorum‐sensing, which plays a critical role in shaping staphylococcal interactions. Through the production and detection of signaling peptides, staphylococci coordinate population‐dependent behaviors and modify gene expression. These quorum‐sensing‐driven transcriptional changes can activate or repress pathways that influence metabolic activity, morphology, motility, aggregation, and interspecies interactions, ultimately shaping the behavior and competitive fitness of both *S. aureus* and co‐colonizing species [63, 64, 65, 66].

In addition, the higher biomass observed in most *S. aureus* and *S. lugdunensis* combinations was accompanied by reduced antibiotic susceptibility. This might be related to the limited penetration capacity of antibiotics into the thicker polymicrobial biofilm communities compared with mono‐species cultures. In line with this study, Harriott and Noverr demonstrated a combination of *S. aureus* and *C. albicans* forms robust polymicrobial biofilms, where the fungal hyphal matrix forms a structural scaffold that boosts bacterial biofilm formation and confers vancomycin resistance to the bacteria [67]. Likewise, *Pseudomonas aeruginosa* and *S. aureus* dual‐species biofilms in a wound‐mimetic environment matured twice as fast and showed an increased antibiotic tolerance by 50% due to diminished antimicrobial penetration capacity, resulting in greater impairment of wound healing compared with mono‐species biofilms [68].

Furthermore, *S. aureus* monocultures reduced HNEC viability more markedly than *S. epidermidis* monocultures, indicating that *S. aureus* is inherently more cytotoxic as a result of the plethora of virulence factors contributing to the success of *S. aureus* as a pathogen [59]. However, *S. aureus*/*S. epidermidis* mixed biofilm resulted in higher HNEC viability than *S. aureus* monoculture, suggesting that *S. epidermidis* partially mitigates *S. aureus* induced cytotoxicity. The lack of a significant difference between *S. epidermidis* monoculture and the coculture indicates that *S. epidermidis* largely determines the overall cytotoxic effect in the mixed biofilm, potentially by buffering or protecting epithelial cells from *S. aureus*‐mediated damage. Thus, while *S. aureus* alone is highly cytotoxic, its coexistence with *S. epidermidis* might attenuate this effect. This might be due to antagonistic interactions between *S. aureus* and *S. epidermidis*, where *S. epidermidis* degrades proteins critical for *S. aureus* biofilm formation and its interaction with the host [69, 70].

In contrast, the viability of HNECs following exposure to *S. aureus* monoculture, *S. lugdunensis* monoculture, or *S. aureus*/*S. lugdunensis* coculture was similarly low, indicating substantial cytotoxicity across all conditions and that coculturing does not amplify or mitigate cell death. These results indicate that both species are highly damaging to epithelial cells, and unlike the *S. aureus*/*S. epidermidis* combination, their coexistence does not confer a protective effect but may instead exacerbate epithelial injury, likely due to synergistic or shared pathogenic mechanisms. This could be attributed to the shared characteristics between *S. aureus* and *S. lugdunensis*, especially their virulence factors, which may influence host response [71].

Furthermore, in *S. aureus*/*S. lugdunensis* pairs but not in *S. aureus*/*S. epidermidis* pairs, high IL‐6 levels were induced in monoculture and mixed biofilm treated HNECs, suggesting a higher pro‐inflammatory potential. This might be due to the niche‐adapted isolates, which might coordinate to express virulence factors at higher levels [72]. Our findings also showed that *S. lugdunensis* biofilm induced greater IL‐6 levels than the *S. epidermidis* biofilm in a single species form. Similar results have been reported by Ramezanpour et al. [15].

One limitation of this study is the small sample size; only three patients with co‐isolation of *S. aureus* and *S. epidermidis*, and three additional patients with co‐isolation of *S. aureus* and *S. lugdunensis*, were included. Therefore, future studies with larger sample sizes are required to better understand the implications of polymicrobial biofilm interactions among these bacteria and their clinical relevance in CRS. Moreover, the genomic landscape of these bacteria and the transcriptomic pathways associated with their interactions were not explored. Future studies should incorporate genomic and transcriptomic analyses to provide a more comprehensive and mechanistic understanding of how these polymicrobial interactions contribute to CRS pathogenesis.

In conclusion, the nature of polymicrobial biofilm interactions and strain‐dependent host adaptation can influence biofilm formation, bacterial virulence, inflammatory potential, and antibiotic responsiveness, all of which have important implications for CRS. Biofilm formation and the inflammatory response of *Staphylococcus* species depend on their co‐colonizing partner. Though *S. aureus* monoculture by itself is cytotoxic, when paired with *S. epidermidis*, the resulting cytotoxicity and inflammation are relatively mild, whereas pairing with *S. lugdunensis* is associated with higher cytotoxicity and IL‐6 induction in both coculture and monoculture forms. Further, the presence of *S. aureus* can influence the biofilm forming capacity of *S. epidermidis* and *S. lugdunensis* and reduce their antimicrobial sensitivity. This study underlines that interspecies interactions among patient‐derived *Staphylococcus* isolates shape the biofilm, cytotoxic, inflammatory, and treatment outcomes that underscore the importance of species‐specific relationships in modulating host responses.

## Author Contributions

The study was designed by S.A. under the supervision of S.V., K.A.F., and A.J.P. S.A performed all experiments, analyzed and interpreted the data, and drafted the manuscript. M.R., C.M.C., M.A., S.F., P.‐J.W, S.V., K.A.F., and A.J.P. provided guidance on methodology, interpretation of results, and manuscript revision. All authors reviewed and approved the final manuscript.

## Funding

This work was supported by the Australian National Health and Medical Research Council (NHMRC) (Grant No. 0006008606) to P.‐J.W., by the University of Adelaide Research Scholarship to S.A., and the Passe and Williams Foundation Senior Fellowship to S.V.

## Conflicts of Interest

The authors declare no conflicts of interest

## Supporting information




**Supporting File 1**: alr70148‐sup‐0001‐tableS1.docx

**Supporting File 1**: alr70148‐sup‐0002‐SuppMat.docx

## References

[1] T. A. Taylor , E. H. Tobin , and C. G. Unakal , Staphylococcus aureus Infection (StatPearls Publishing, 2017). 28722898

[2] B. Maddiboyina , H. Roy , M. Ramaiah , et al., “Methicillin‐Resistant *Staphylococcus aureus*: Novel Treatment Approach Breakthroughs,” Bulletin of the National Research Centre 47, no. 1 (2023): 95.

[3] M. Goetghebeur , P.‐A. Landry , D. Han , and C. Vicente , “Methicillin‐Resistant *Staphylococcus aureus*: A Public Health Issue With Economic Consequences,” Canadian Journal of Infectious Diseases and Medical Microbiology 18, no. 1 (2007): 27–34. 18923684 10.1155/2007/253947 PMC2542887

[4] S. S. Adeiza and I. Aminul , “Meta‐Meta‐Analysis of the Mortality Risk Associated With MRSA Compared to MSSA Bacteraemia,” Le Infezioni in Medicina 32, no. 2 (2024): 131. 38827830 10.53854/liim-3202-2 PMC11142415

[5] Z. Bendouah , J. Barbeau , W. A. Hamad , and M. Desrosiers , “Biofilm Formation by *Staphylococcus aureus* and *Pseudomonas aeruginosa* Is Associated With an Unfavorable Evolution After Surgery for Chronic Sinusitis and Nasal Polyposis,” Otolaryngology—Head and Neck Surgery 134, no. 6 (2006): 991–996. 16730544 10.1016/j.otohns.2006.03.001

[6] G. Shaghayegh , C. Cooksley , G. Bouras , et al., “ *Staphylococcus aureus* Biofilm Properties and Chronic Rhinosinusitis Severity Scores Correlate Positively With Total CD4+ T‐cell Frequencies and Inversely With Its Th1, Th17 and Regulatory Cell Frequencies,” Immunology 170, no. 1 (2023): 120–133. 37191458 10.1111/imm.13655

[7] T. J. Foster , “ *Staphylococcus aureus* ,” in Molecular Medical Microbiology, ed. M. Sussman (Academic Press, 2002), 839–888.

[8] J. P. Hymes and T. R. Klaenhammer , “Stuck in the Middle: Fibronectin‐Binding Proteins in Gram‐Positive Bacteria,” Frontiers in Microbiology 7 (2016): 1504. 27713740 10.3389/fmicb.2016.01504 PMC5031765

[9] G. Shaghayegh , C. Cooksley , M. Ramezanpour , P.‐J. Wormald , A. J. Psaltis , and S. Vreugde , “Chronic Rhinosinusitis, *S. aureus* Biofilm and Secreted Products, Inflammatory Responses, and Disease Severity,” Biomedicines 10, no. 6 (2022): 1362. 35740385 10.3390/biomedicines10061362 PMC9220248

[10] K. Tam and V. Torres , “ *Staphylococcus aureus* Secreted Toxins & Extracellular Enzymes Kayan,” Physiology & Behavior 176 (2016): 139–148.

[11] A. J. McCarthy , A. Loeffler , A. A. Witney , K. A. Gould , D. H. Lloyd , and J. A. Lindsay , “Extensive Horizontal Gene Transfer During *Staphylococcus aureus* Co‐Colonization In Vivo,” Genome Biology and Evolution 6, no. 10 (2014): 2697–2708. 25260585 10.1093/gbe/evu214 PMC4224341

[12] J. T. Smith and C. P. Andam , “Extensive Horizontal Gene Transfer Within and Between Species of Coagulase‐Negative *Staphylococcus* ,” Genome Biology and Evolution 13, no. 9 (2021): evab206. 34498042 10.1093/gbe/evab206 PMC8462280

[13] M. Michalik , A. Samet , A. Podbielska‐Kubera , V. Savini , J. Międzobrodzki , and M. Kosecka‐Strojek , “Coagulase‐Negative Staphylococci (CoNS) as a Significant Etiological Factor of Laryngological Infections: A Review,” Annals of Clinical Microbiology and Antimicrobials 19 (2020): 1–10. 32498711 10.1186/s12941-020-00367-x PMC7271473

[14] Z. Zhang , N. D. Adappa , E. Lautenbach , et al., eds., Coagulase‐Negative Staphylococcus Culture in Chronic Rhinosinusitis (International Forum of Allergy & Rhinology: Wiley Online Library, 2015). 10.1002/alr.21439 PMC4352125 25367456

[15] M. Ramezanpour , S. Feizi , H. D. P. Arachchige , et al., eds. Evaluation of Mucosal Barrier Disruption Due to Staphylococcus lugdunensis and Staphylococcus epidermidis Exoproteins in Patients With Chronic Rhinosinusitis (International Forum of Allergy & Rhinology: Wiley Online Library, 2025). 10.1002/alr.23481 39512125

[16] M. Michalik , A. Nowakiewicz , A. Trościańczyk , C. Kowalski , and A. Podbielska‐Kubera , “Multidrug Resistant Coagulase‐Negative *Staphylococcus* spp. Isolated From Cases of Chronic Rhinosinusitis in Humans. Study From Poland,” Acta Microbiologica et Immunologica Hungarica 69, no. 1 (2022): 68–76. 34898473 10.1556/030.2021.01580

[17] M. Otto , “ *Staphylococcus epidermidis*—The 'accidental' pathogen,” Nature Reviews Microbiology 7, no. 8 (2009): 555–567. 19609257 10.1038/nrmicro2182 PMC2807625

[18] S. S. Dastgheyb , A. E. Villaruz , K. Y. Le , et al., “Role of Phenol‐Soluble Modulins in Formation of *Staphylococcus aureus* Biofilms in Synovial Fluid,” Infection and Immunity 83, no. 7 (2015): 2966–2975. 25964472 10.1128/IAI.00394-15 PMC4468530

[19] K. Y. Le , A. E. Villaruz , Y. Zheng , et al., “Role of Phenol‐Soluble Modulins in *Staphylococcus epidermidis* Biofilm Formation and Infection of Indwelling Medical Devices,” Journal of Molecular Biology 431, no. 16 (2019): 3015–3027. 30954574 10.1016/j.jmb.2019.03.030 PMC10999989

[20] P. D. Fey and M. E. Olson , “Current Concepts in Biofilm Formation of *Staphylococcus epidermidis* ,” Future Microbiology 5, no. 6 (2010): 917–933. 20521936 10.2217/fmb.10.56 PMC2903046

[21] Q. Liu , Q. Liu , H. Meng , et al., “ *Staphylococcus epidermidis* Contributes to Healthy Maturation of the Nasal Microbiome by Stimulating Antimicrobial Peptide Production,” Cell Host & Microbe 27, no. 1 (2020): 68.e5–78.e5. 31866425 10.1016/j.chom.2019.11.003 PMC10988655

[22] S. Heilbronner and T. J. Foster , “ *Staphylococcus lugdunensis*: A Skin Commensal With Invasive Pathogenic Potential,” Clinical Microbiology Reviews 34, no. 2 (2020): e00205‐20. 33361142 10.1128/CMR.00205-20 PMC7950365

[23] J. Lourtet‐Hascoët , A. Bicart‐See , M. Félicé , G. Giordano , and E. Bonnet , “ *Staphylococcus lugdunensis*, a Serious Pathogen in Periprosthetic Joint Infections: Comparison to *Staphylococcus aureus* and *Staphylococcus epidermidis* ,” International Journal of Infectious Diseases 51 (2016): 56–61. 27609028 10.1016/j.ijid.2016.08.007

[24] N. K. Archer , M. J. Mazaitis , J. W. Costerton , J. G. Leid , M. E. Powers , and M. E. Shirtliff , “ *Staphylococcus aureus* Biofilms: Properties, Regulation, and Roles in Human Disease,” Virulence 2, no. 5 (2011): 445–459. 21921685 10.4161/viru.2.5.17724 PMC3322633

[25] J. A. Lindsay and M. T. Holden , “ *Staphylococcus aureus*: Superbug, Super Genome?,” Trends in Microbiology 12, no. 8 (2004): 378–385. 15276614 10.1016/j.tim.2004.06.004

[26] S. T. Aka and S. H. Haji , “Sub‐MIC of Antibiotics Induced Biofilm Formation of *Pseudomonas aeruginosa* in the Presence of Chlorhexidine,” Brazilian Journal of Microbiology 46 (2015): 149–154. 26221101 10.1590/S1517-838246120140218 PMC4512058

[27] J. Wille and T. Coenye , “Biofilm Dispersion: The Key to Biofilm Eradication or Opening Pandora's Box?,” Biofilm 2 (2020): 100027. 33447812 10.1016/j.bioflm.2020.100027 PMC7798462

[28] C. W. Hall and T.‐F. Mah , “Molecular Mechanisms of Biofilm‐Based Antibiotic Resistance and Tolerance in Pathogenic Bacteria,” FEMS Microbiology Reviews 41, no. 3 (2017): 276–301. 28369412 10.1093/femsre/fux010

[29] E. Y. Trizna , M. N. Yarullina , D. R. Baidamshina , et al., “Bidirectional Alterations in Antibiotics Susceptibility in *Staphylococcus aureus*—*Pseudomonas aeruginosa* Dual‐Species Biofilm,” Scientific Reports 10, no. 1 (2020): 14849. 32908166 10.1038/s41598-020-71834-w PMC7481796

[30] M. Pammi , R. Liang , J. Hicks , T.‐A. Mistretta , and J. Versalovic , “Biofilm Extracellular DNA Enhances Mixed Species Biofilms of *Staphylococcus epidermidis* and *Candida albicans* ,” BMC Microbiology 13 (2013): 1–13. 24228850 10.1186/1471-2180-13-257 PMC3833181

[31] A. V. Mironova , A. V. Karimova , M. I. Bogachev , A. R. Kayumov , and E. Y. Trizna , “Alterations in Antibiotic Susceptibility of *Staphylococcus aureus* and *Klebsiella pneumoniae* in Dual Species Biofilms,” International Journal of Molecular Sciences 24, no. 10 (2023): 8475. 37239822 10.3390/ijms24108475 PMC10217825

[32] S. González , L. Fernández , A. B. Campelo , et al., “The Behavior of *Staphylococcus aureus* Dual‐Species Biofilms Treated With Bacteriophage phiIPLA‐RODI Depends on the Accompanying Microorganism,” Applied and Environmental Microbiology 83, no. 3 (2017): e02821–16. 27836851 10.1128/AEM.02821-16 PMC5244312

[33] B. R. H. Cervantes‐Huamán , C. Ripolles‐Avila , T. Mazaheri , and J. J. Rodríguez‐Jerez , “Pathogenic Mono‐Species Biofilm Formation on Stainless Steel Surfaces: Quantitative, Qualitative, and Compositional Study,” LWT 159 (2022): 113211.

[34] C. Jardeleza , A. Foreman , L. Baker , et al., eds., The Effects of Nitric Oxide on Staphylococcus aureus Biofilm Growth and Its Implications in Chronic Rhinosinusitis (International Forum of Allergy & Rhinology, Wiley Online Library, 2011). 10.1002/alr.20083 22144052

[35] A. A. Prince , J. D. Steiger , A. N. Khalid , et al., “Prevalence of Biofilm‐Forming Bacteria in Chronic Rhinosinusitis,” American Journal of Rhinology 22, no. 3 (2008): 239–245. 18588755 10.2500/ajr.2008.22.3180

[36] S. Oncel , E. Pinar , G. Sener , C. Calli , and U. Karagoz , “Evaluation of Bacterial Biofilms in Chronic Rhinosinusitis,” Journal of Otolaryngology–Head & Neck Surgery 39, no. 1 (2010): 52–55. 20122345

[37] A. Foreman , G. Holtappels , A. Psaltis , et al., “Adaptive Immune Responses in *Staphylococcus aureus* Biofilm–Associated Chronic Rhinosinusitis,” Allergy 66, no. 11 (2011): 1449–1456. 21834937 10.1111/j.1398-9995.2011.02678.x

[38] W. J. Fokkens , V. J. Lund , J. Mullol , et al., “EPOS 2012: European Position Paper on Rhinosinusitis and Nasal Polyps 2012. A Summary for Otorhinolaryngologists,” Rhinology 50, no. 1 (2012): 1–12. 22469599 10.4193/Rhino12.000

[39] S. C. Payne , M. McKenna , J. Buckley , et al., “Clinical Practice Guideline: Adult Sinusitis Update,” Otolaryngology–Head and Neck Surgery 173 (2025): S1–S56. 40742114 10.1002/ohn.1344

[40] V. J. Lund and I. S. Mackay , “Staging in Rhinosinusitis,” Rhinology 31 (1993): 183. 8140385

[41] V. J. Lund and D. W. Kennedy , “Staging for Rhinosinusitis,” Otolaryngology–Head and Neck Surgery 117, no. 3 (1997): S35–S40.9334786

[42] A. J. Psaltis , G. Li , R. Vaezeafshar , K. S. Cho , and P. H. Hwang , “Modification of the Lund‐Kennedy Endoscopic Scoring System Improves Its Reliability and Correlation With Patient‐Reported Outcome Measures,” Laryngoscope 124, no. 10 (2014): 2216–2223. 24615873 10.1002/lary.24654

[43] C. Hopkins , S. Gillett , R. Slack , V. Lund , and J. Browne , “Psychometric Validity of the 22‐Item Sinonasal Outcome Test,” Clinical Otolaryngology 34, no. 5 (2009): 447–454. 19793277 10.1111/j.1749-4486.2009.01995.x

[44] G. Shaghayegh , C. Cooksley , G. Bouras , et al., “ *S. aureus* Biofilm Properties Correlate With Immune B Cell Subset Frequencies and Severity of Chronic Rhinosinusitis,” Clinical Immunology 263, (2024): 263. 10.1016/j.clim.2024.110221 38636891

[45] G. Houtak , G. Bouras , R. Nepal , et al., “The Intra‐Host Evolutionary Landscape and Pathoadaptation of Persistent *Staphylococcus aureus* in Chronic Rhinosinusitis,” Microbial Genomics 9, no. 11 (2023): 001128. 38010322 10.1099/mgen.0.001128 PMC10711304

[46] M. Ramezanpour , H. Bolt , A. Psaltis , P. J. Wormald , and S. Vreugde , “Inducing a Mucosal Barrier–Sparing Inflammatory Response in Laboratory‐Grown Primary Human Nasal Epithelial Cells,” Current Protocols in Toxicology 80, no. 1 (2019): e69. 30715797 10.1002/cptx.69

[47] S. Toma and C. Hopkins , “Stratification of SNOT‐22 Scores Into Mild, Moderate or Severe and Relationship With Other Subjective Instruments,” Rhinology 54, no. 2 (2016): 129–133. 27017484 10.4193/Rhino15.072

[48] C. A. Lux , B. Wagner Mackenzie , J. Johnston , et al., “Antibiotic Treatment for Chronic Rhinosinusitis: Prescription Patterns and Associations With Patient Outcome and the Sinus Microbiota,” Frontiers in Microbiology 11 (2020): 595555. 33414772 10.3389/fmicb.2020.595555 PMC7782326

[49] S. Águila‐Arcos , I. Álvarez‐Rodríguez , O. Garaiyurrebaso , C. Garbisu , E. Grohmann , and I. Alkorta , “Biofilm‐Forming Clinical *Staphylococcus* Isolates Harbor Horizontal Transfer and Antibiotic Resistance Genes,” Frontiers in Microbiology 8 (2017): 2018. 29085354 10.3389/fmicb.2017.02018 PMC5650641

[50] B. Mirzaei , P. Faridifar , M. Shahmoradi , et al., “Genotypic and Phenotypic Analysis of Biofilm Formation *Staphylococcus epidermidis* Isolates From Clinical Specimens,” BMC Research Notes 13, no. 1 (2020): 114. 32103775 10.1186/s13104-020-04965-y PMC7045379

[51] H. Büttner , D. Mack , and H. Rohde , “Structural Basis of *Staphylococcus epidermidis* Biofilm Formation: Mechanisms and Molecular Interactions,” Frontiers in Cellular and Infection Microbiology 5 (2015): 14. 25741476 10.3389/fcimb.2015.00014 PMC4330918

[52] Q. Peng , X. Tang , W. Dong , N. Sun , and W. Yuan , “A Review of Biofilm Formation of *Staphylococcus aureus* and Its Regulation Mechanism,” Antibiotics 12, no. 1 (2022): 12. 36671212 10.3390/antibiotics12010012 PMC9854888

[53] J. S. Madsen , M. Burmølle , L. H. Hansen , and S. J. Sørensen , “The Interconnection Between Biofilm Formation and Horizontal Gene Transfer,” FEMS Immunology & Medical Microbiology 65, no. 2 (2012): 183–195. 22444301 10.1111/j.1574-695X.2012.00960.x

[54] E. Giaouris , E. Heir , M. Desvaux , et al., “Intra‐and Inter‐Species Interactions Within Biofilms of Important Foodborne Bacterial Pathogens,” Frontiers in Microbiology 6 (2015): 841. 26347727 10.3389/fmicb.2015.00841 PMC4542319

[55] C. Beloin and J.‐M. Ghigo , “Finding Gene‐Expression Patterns in Bacterial Biofilms,” Trends in Microbiology 13, no. 1 (2005): 16–19. 15639627 10.1016/j.tim.2004.11.008

[56] R. Moonsamy , "Investigating the Effect of *Staphylococcus epidermidis* on the Growth Dynamics of *Staphylococcus aureus* in Atopic Dermatitis" (master's thesis, University of Cape Town, 2025).

[57] A. M. S. Figueiredo , F. A. Ferreira , C. O. Beltrame , and M. F. Côrtes , “The Role of Biofilms in Persistent Infections and Factors Involved in Ica‐Independent Biofilm Development and Gene Regulation in *Staphylococcus aureus* ,” Critical Reviews in Microbiology 43, no. 5 (2017): 602–620. 28581360 10.1080/1040841X.2017.1282941

[58] J. Valle , A. Toledo‐Arana , C. Berasain , et al., “SarA and Not σB Is Essential for Biofilm Development by *Staphylococcus aureus* ,” Molecular Microbiology 48, no. 4 (2003): 1075–1087. 12753197 10.1046/j.1365-2958.2003.03493.x

[59] K. Tam and V. J. Torres , “ *Staphylococcus aureus* Secreted Toxins and Extracellular Enzymes,” Microbiology Spectrum 7, no. 2 (2019), 10.1128/microbiolspec.gpp3-0039-2018. PMC6422052 30873936

[60] C. O. Olwal , P. O. Ang'ienda , and D. O. Ochiel , “Alternative Sigma Factor B (σB) and Catalase Enzyme Contribute to *Staphylococcus epidermidis* Biofilm's Tolerance Against Physico‐Chemical Disinfection,” Scientific Reports 9, no. 1 (2019): 5355. 30926870 10.1038/s41598-019-41797-8 PMC6440968

[61] I. Vandecandelaere , F. Van Nieuwerburgh , D. Deforce , and T. Coenye , “Metabolic Activity, Urease Production, Antibiotic Resistance and Virulence in Dual Species Biofilms of *Staphylococcus epidermidis* and *Staphylococcus aureus* ,” PLoS ONE 12, no. 3 (2017): e0172700. 28263995 10.1371/journal.pone.0172700 PMC5338783

[62] G. Houtak , G. Bouras , R. Nepal , et al., “The Intra‐Host Evolutionary Landscape and Pathoadaptation of Persistent *Staphylococcus aureus* in Chronic Rhinosinusitis,” Microbial Genomics 9, no. 11 (2023): 001128. 38010322 10.1099/mgen.0.001128 PMC10711304

[63] J. Canovas , M. Baldry , M. S. Bojer , et al., “Cross‐Talk Between *Staphylococcus aureus* and Other Staphylococcal Species via the Agr Quorum Sensing System,” Frontiers in Microbiology 7 (2016): 1733. 27877157 10.3389/fmicb.2016.01733 PMC5099252

[64] M. J. Federle and B. L. Bassler , “Interspecies Communication in Bacteria,” The Journal of Clinical Investigation 112, no. 9 (2003): 1291–1299. 14597753 10.1172/JCI20195 PMC228483

[65] L. Laganenka and V. Sourjik , “Bacterial Quorum Sensing Signals at the Interdomain Interface,” Israel Journal of Chemistry 63, no. 5–6 (2023): e202200080.

[66] M. Li , G. Zhao , and M. M. Li , “Regulatory Mechanisms of Quorum Sensing in Microbial Communities and Their Potential Applications in Ruminant Livestock Production,” Journal of Advanced Research (2025), 10.1016/j.jare.2025.07.055. PMC13131436 40749791

[67] M. M. Harriott and M. C. Noverr , “ *Candida albicans* and *Staphylococcus aureus* Form Polymicrobial Biofilms: Effects on Antimicrobial Resistance,” Antimicrobial Agents and Chemotherapy 53, no. 9 (2009): 3914–3922. 19564370 10.1128/AAC.00657-09 PMC2737866

[68] P. K. Vestweber , J. Wächter , V. Planz , N. Jung , and M. Windbergs , “The Interplay of *Pseudomonas aeruginosa* and *Staphylococcus aureus* in Dual‐Species Biofilms Impacts Development, Antibiotic Resistance and Virulence of Biofilms in in Vitro Wound Infection Models,” PLoS ONE 19, no. 5 (2024): e0304491. 38805522 10.1371/journal.pone.0304491 PMC11132468

[69] S. Sugimoto , T. Iwamoto , K. Takada , et al., “ *Staphylococcus epidermidis* Esp Degrades Specific Proteins Associated With *Staphylococcus aureus* Biofilm Formation and Host‐Pathogen Interaction,” Journal of Bacteriology 195, no. 8 (2013): 1645–1655. 23316041 10.1128/JB.01672-12 PMC3624567

[70] T. Iwase , Y. Uehara , H. Shinji , et al., “ *Staphylococcus epidermidis* Esp Inhibits *Staphylococcus aureus* Biofilm Formation and Nasal Colonization,” Nature 465, no. 7296 (2010): 346–349. 20485435 10.1038/nature09074

[71] S. Ravaioli , D. Campoccia , R. Mirzaei , et al., “Searching for Virulence Factors Among *Staphylococcus lugdunensis* Isolates From Orthopedic Infections: Correlation of β‐Hemolysin, Hemolysin III, and Slush Genes With Hemolytic Activity and Synergistic Hemolytic Activity,” International Journal of Molecular Sciences 24, no. 21 (2023): 15724. 37958706 10.3390/ijms242115724 PMC10650139

[72] S. S. Dastgheyb and M. Otto , “Staphylococcal Adaptation to Diverse Physiologic Niches: An Overview of Transcriptomic and Phenotypic Changes in Different Biological Environments,” Future Microbiology 10, no. 12 (2015): 1981–1995. 26584249 10.2217/fmb.15.116 PMC4946774

