# Supplementary material for: Interspecies Biofilm Dynamics Among Staphylococci: Inflammatory Contributions to Chronic Rhinosinusitis
Source: Int Forum Allergy Rhinol. 2026 Mar 31;16(9):956–67. doi: 10.1002/alr.70148 (PMC13544510; doi:10.1002/alr.70148)
Supplement: Supplementary file 1 [file ALR-16-956-s001.docx]

**Supplementary Figures**

**Supplementary Figure 1: Biofilm colony-forming unit (CFU) value of *S. aureus* (SA), *S. epidermidis* (SE) *and S. lugdunensis* (SL) cultured singly or in coculture (SA + SE; SA + SL) for patients 1-6. (**A- C) represent isolates from patients 1- 3, whereas (D- F) represent isolates from patients 4- 6, respectively. Results are in log CFU/mL and represented as mean ± SEM. * p ≤0.05, ** p ≤0.01, ***p ≤ 0.001, **** p ≤ 0.0001; ns: not statistically significant (One-way Anova followed by Tukey's multiple comparisons test)*.* N= 3 replicates

**Supplementary Figure 2: Biofilm biomass value (OD_595_ nm) of *S. aureus* (SA) and *S. lugdunensis* (SL) in monoculture and coculture from different patients using Transwell plates.** Patient-derived isolates are indicated by numbers (4, 5, 6). In coculture, the first species in the label represents the biomass value grown in the lower chamber, and the second species represents the bacteria in the upper chamber (e.g., SL–SA: SL in the bottom and SA in the upper chamber; SA–SL: SA in the bottom and SL in the upper chamber). Data are presented as mean ± SEM. ns = not statistically significant.
