# Supplementary material for: Interspecies Biofilm Dynamics Among Staphylococci: Inflammatory Contributions to Chronic Rhinosinusitis
Source: Int Forum Allergy Rhinol. 2026 Mar 31;16(9):956–67. doi: 10.1002/alr.70148 (PMC13544510; doi:10.1002/alr.70148)
Supplement: Supplementary file 2 [file ALR-16-956-s002.docx]

**Supplementary Table 1: Minimum Inhibitory Concentration (MIC (mg/L)) values of Amoxicillin and Doxycycline against *S. aureus, S. epidermidis* and *S. lugdunensis* strains**

| **Patients** | **Clinical isolates** | **AMX MIC value (mg/L)** | **Doxycycline MIC value(mg/L)** |
| --- | --- | --- | --- |
| Patient 1 | *S. aureus (*SA1) | 2 | 0.25 |
|  | *S. epidermidis* (SE1) | 2 | 0.5 |
| Patient 2 | *S. aureus* (SA2) | 4 | 0.5 |
|  | *S. epidermidis* (SE2) | 2 | 0.25 |
| Patient 3 | *S. aureus* (SA3) | 0.5 | 0.25 |
|  | *S. epidermidis* (SE3) | 0.25 | 0.25 |
| Patient 4 | *S. aureus* (SA4) | 1 | 0.5 |
|  | *S. lugdunensis* (SL4) | 1 | 0.5 |
| Patient 5 | *S. aureus* (SA5) | 2 | 0.5 |
|  | *S. lugdunensis* (SL5) | 1 | 1 |
| Patient 6 | *S. aureus* (SA6) | 2 | 1 |
|  | *S. lugdunensis* (SL6) | 1 | 0.5 |

SA: *S. aureus*, SE: *S. epidermidis,* SL*: S. lugdunensis*
